# Social networks, health-promoting behaviors, and health-related quality of life in older adults with and without arthritis

**DOI:** 10.1371/journal.pone.0220180

**Published:** 2019-07-24

**Authors:** Minjoo Hong, Hyewon Shin, Jennie C. De Gagne

**Affiliations:** 1 Department of Nursing, Gyeongnam National University of Science and Technology, Jinju, South Korea; 2 School of Nursing, Clemson University, Greenville, South Carolina, United States of America; 3 School of Nursing, Duke University, Durham, North Carolina, United States of America; Indiana University Purdue University at Indianapolis, UNITED STATES

## Abstract

The purpose of this secondary analysis study was to compare social networks, health-promoting behaviors, and health-related quality of life of South Korean adults, aged 65 years or older, with and without arthritis, and to identify factors that are related to health-related quality of life. The sample consisted of 103 adults with arthritis and 123 adults without arthritis. Data were analyzed using a two-way analysis of variance, *χ*^2^-test, Pearson’s correlation, and multiple regression analysis. All variables except age and religion showed statistically significant differences between older adults with and without arthritis. The group with arthritis reported lower scores on social networks, health-promoting behaviors, and health-related quality of life compared to the healthy group without arthritis. Analyzed using multiple regression, 43.8% of the older adults with arthritis had the factors related to health-related quality of life (*F* = 40.71, *p* < .001) including exercise (β = .43, *p* < .001) and living with someone (β = .32, *p* = .001). In the group of older adults without arthritis, 26.2% had the factors related to health-related quality of life (*F* = 15.44, *p* < .001) including exercise (β = .31, *p =* .001), social gatherings, and employment status. Exercise was one of the factors that showed the strongest relationship to health-related quality of life. The provision of resources that can enable an individual to engage in physical activities is warranted.

## Introduction

South Korea has entered the league of aging societies, with those aged 65 or older accounting for more than 14% of the total population. In 2025, it is expected to become a super-aged society where the older adult population will account for more than 20% of the total population [1]. Along with the increase in life expectancy, the prevalence of chronic diseases in older adults is also increasing. According to a Korean national survey, the proportion of Korean older adults diagnosed with one or more chronic diseases was 89.5%, and adults with two or more diseases was 73%, showing a continuous increase from the beginning of the survey from 2008 to 2017 [2]. Thus, it is necessary to continually review measures to maintain and improve the health-related quality of life (HRQOL) of older adults with chronic diseases. The United Nations has agreed that “older than 65 years” denotes “old age” in their World Population Ageing report standard about old age and the World Health Organization’s report [3].

Arthritis is a representative chronic disease that can, directly and indirectly, influence the HRQOL of older adults because it is difficult to completely cure while symptoms tend to get worse as aging progresses. The prevalence of arthritis in older adults in Korea is 33%, making it the second most prevalent chronic disease after hypertension [2]; and arthritis in the United States (U.S.) accounts for about 29% of chronic diseases [4]. According to the findings in the U.S. report, well-managed arthritis is associated with better HRQOL [5] in arthritis patients because inactivity due to arthritis causes considerable emotional stress and limits social activities, and in many cases is accompanied by other chronic diseases [6,7]. Because Korean older adults often consider arthritis as a natural process of aging, they do not get proper treatment and frequently experience a poorer quality of life [8]. Thus, it is essential to identify those disease conditions which are related to HRQOL by comparing older adults with and without arthritis to provide the necessary interventions.

To understand the HRQOL of older adults, examining their environmental factors is vital. One of the important environmental factors that affect the successful aging of older adults is a social network [9,10]. Korean older adults tend to obtain health-related information from friends or family before seeing a doctor [11]. In this context, it is necessary to determine the social network of family and friends and the disease conditions on HRQOL by comparing older adults with and without arthritis. In this paper, the term “social network” refers to social support and social activities the individual has with their family and friends. We measured social network using LSNS-6 which asks about the number of relatives and friends in order to measure how often they contact each other, feel close enough to request for help, and feel at ease enough to share about private matters [12].

A social network can lower the death risk of older adults, positively affect their quality of life [13], and provide the information or motivation necessary for health-promoting behaviors. For that reason, before attempting to improve the HRQOL of arthritic older adults with the limiting factors of social activities and physical functions, it is imperative to identify their social networks.

The symptoms of arthritis, including pain and ankylosis that can lower HRQOL, need to be controlled by health-promoting behaviors [14]. It is difficult, however, for older adults with arthritis to maintain health-promoting behaviors such as regular exercise [15]. Health-promoting behaviors are frequently investigated as being relevant to the HRQOL of older adults with arthritis because these behaviors can help older adults to maintain functions independently [16], and are more likely to change than fixed factors such as environmental factors or economic level [17]. Since frequent assessment of the influence of arthritis on HRQOL and its proper management are necessary [18], identifying the relationship between health-promoting behaviors and social network, and searching for activity plans related to these variables are essential for the improvement of the HRQOL of older adults with arthritis.

The quality of life of older adults with musculoskeletal diseases has been reported to be significantly poorer than that of older adults without such diseases [5,18]. Research on HRQOL of older adults has used general characteristics such as gender, age, educational level, marital status, personal income level, socioeconomic characteristics, or health-related characteristics such as health condition or health behavior as the independent variables [8,17]. Research has found that for older adults with chronic illnesses, such as cardiovascular disease, heart disease, cancer, cognitive disorder, and dementia, when their social network is sufficient, the network can play a role in living a healthy life with a chronic illness [10,13]. There is, however, an insufficient amount of research on patients with arthritis who have good social networks and HRQOL compared to research on other chronic illnesses.

The identification of social networks, health-promoting behaviors, and HRQOL in a specific group can provide valuable information essential for predicting the health trends of the group [10]. A comparative study between groups with and without diseases offers the advantage of accurately determining the factors associated with the dependent variables [19]. The present study aims to identify factors associated with HRQOL in older adults with arthritis, focusing on social networks with family and friends and health-promoting behaviors. We hypothesized that the social networks of people with arthritis are similar to of people who do not have arthritis. According to the Korean national survey [20], older adults with chronic illness reported that their family support had impacted on their quality of life regardless of chronic illness such as arthritis. Second, we hypothesized that older adults with arthritis report less health-promoting behaviors than older adults without arthritis. Third, we hypothesized that older adults with arthritis report lower HRQOL scores than older adults without arthritis.

The purpose of the present study was to analyze the general characteristics of older adults, social networks, health-promoting behaviors, and HRQOL related to the presence of arthritis, and compare the factors that are associated with HRQOL. The specific goals were to identify and compare the demographic characteristics of older adults with and without arthritis, comparing the social networks of family and friends, health-promoting behaviors, and levels of HRQOL between the two groups; and thus, identify the factors associated with HRQOL in older adults with arthritis, focusing on social networks and health-promotion behaviors.

## Materials and methods

A secondary analysis was performed for the current study using data from the study by Hong et al. [10] that compared HRQOL between older adults residing in Korea and the immigrants in U.S. The original study [10] was descriptive, and the data included 354 older adults aged 65 years or older residing in their own homes in the Busan and Gyeongnam areas of Korea and North Carolina in the U.S. That study examined the relationship between several sociodemographic characteristics and the HRQOL of each group. Each group’s HRQOL was compared to determine the extent of the relationship between their social network scores and their health-promoting behaviors. The social network, health-related quality of life, and health-promoting behaviors were higher for the older adults who live in U.S. The current study focuses on a vulnerable population, namely older Korean adults with/without arthritis living in South Korea. The sample consists of 226 Korean adults from the Hong et al. data, who are aged 65 years or older, with self-reported arthritis (n = 103) and without arthritis (n = 123). A sample of 212 subjects was necessary for a significance level of 5%, power of test = 80%, and medium effect size for a two-tailed test calculated according to Cohen’s equation using the G*Power 3.1 program.

### Measures

#### General characteristics

The general characteristics of the subjects included age, gender, presence of a cohabiting family, education, frequency of social gatherings, employment, income level, and income source. All variables were categorical.

#### Social network

The abridged version of the Lubben Social Network Scale (LSNS; LSNS-6) [21] was used to assess social network. The LSNS-6 was designed to determine social support and isolation recognized by the family, friends, and neighbors of people who have difficulty responding to long questionnaire items. The questionnaire is composed of a total of six items divided into two factors: family (including relatives) and friends (including neighbors). Each factor is composed of three items and the range of responses is categorized into “0 persons,” “1 person,” “2 people,” “3–4 people,” “5–8 people,” and “9 or more people.” The score is calculated by summing the score of each item, and the maximum score is 30 points. The reliability at the time of development measured by Cronbach's α was .83, and that of the present study was .92.

#### Health-promoting lifestyle profile

A modified and supplemented version of the existing Health Promotion Lifestyle Profile (HPLP; HPLP-II) was used [22,23] to assess the health-promoting lifestyle behaviors. The instrument is composed of 52 items categorized into six sub-domains: spiritual growth, nutrition, health responsibility, exercise, interpersonal relationship, and stress management. Items are rated on a four-point scale from “never” (1 point) to “always” (4 points), and the higher the score, the higher the level of performance of health-promoting behaviors. The reliability of the instrument measured at the time of development using Cronbach's α coefficient was .94, and that of the present study was .97.

#### Health-related quality of life

To measure the overall HRQOL, the official Korean version of the EuroQol 5-Dimension 5-Level [24], was used. C*rosswalk value sets for the EQ-5D-5L* required from the EuroQol website were used. EQ-5D-5L value sets are available for each country that has conducted a valuation study. By using the crosswalk link function and the individual responses to the EQ-5D5L descriptive system, index values for the EQ-5D-5L can be calculated. When the data is input, the score is automatically calculated [24]. It is composed of five items: mobility, self-management, daily activities, pain/discomfort, and anxiety/depression. The participant provides a score (1: no problems, 2: slight problems, 3: moderate problems, 4: severe problems, and 5: extreme problems*)*. An index score ranges from -1 to 1 where an index score of 1 reflects the best possible HRQOL and an index score of <1 is indicative of impaired HRQOL. The reliability of the original instrument measure by Cronbach's α was .75, and the α of the present study was .90.

### Data collection

The criteria for inclusion in the study were that subjects had to be (a) community dwellers in Korea, (b) 65 years of age or older, and (c) able to understand Korean sufficiently to answer the questionnaires. The authors performed a secondary analysis of the primary data after obtaining approval from the Institutional Review Board of the University of North Carolina at Greensboro (#16–0244) in the U.S. The primary research was approved by the Institutional Review Board of the Duke University (Pro00067114) in the U.S. In the primary research, written consent was obtained from all participants. During data collection, the researchers and a research assistant assisted the participants in filling in the survey so participants were able to answer almost all questions minimizing missing data.

### Data analysis method

The collected data were analyzed using SPSS 21.0 for Windows (IBM Corp., Armonk, N.Y., USA). The general characteristics, social networks, health-promoting behaviors, and HRQOL of the subjects were analyzed by frequency, percentage, average, and standard deviation, and between-group differences in general characteristics were analyzed using the chi-square test (χ^2^ test). To determine differences related to the general characteristics, *t*-tests were conducted, and a two-way analysis of variance was used to determine the differences in social networks, health-promoting behaviors, and level of HRQOL related to the general characteristics. Scheffe's test was used for post-hoc analyses, and Pearson’s correlation coefficients were calculated to determine the correlations among variables. Tolerance and variation inflation factor (VIF) were used to determine multicollinearity among the independent variables, and factors associated with HRQOL were analyzed using multiple regression analysis.

## Results

### Differences in characteristics between older adults with arthritis and older adults without arthritis

Statistically significant differences (p < .001) were found between older adults with arthritis and older adults without arthritis across all the general characteristic variables except age. The proportion of subjects living alone was 58.3% for the arthritis group and 41.5% for the group without arthritis. The most prevalent educational level of the arthritis group was no education (35%) while that of the group without arthritis was high school graduation or more (32.5%). Nearly a third (35%) of the subjects in the arthritis group did not have regular meetings such as social gatherings while only 18.7% in the group without arthritis did not have regular meetings. The weekly frequency of social gathering was higher for the no arthritis group than that of the arthritis group. In terms of monthly income, 52.4% of the arthritis group had an income of KRW 500,000 or less, and income from adult children constituted 54.4% of the income source. Only 30.9% of the group without arthritis had a monthly income of KRW 500,000 or less, and 43.5% had earned income (Table 1).

**Table 1:** Characteristics of older adults with arthritis and older adults without arthritis (N = 226).

Characteristics	Categories	Older adults with arthritis (n = 103)	Older adults without arthritis (n = 123)	*χ*^2^	*p*
		n (Column %)	n (Column %)		
Age (years)	≤ = 74	46 (44.7)	60 (48.8)	0.38	0.593
	≥75	57 (55.3)	63 (51.2)		
Gender	Male	21 (20.4)	44 (35.8)	6.47	0.012
	Female	82 (79.6)	79 (64.2)		
Residing	Alone	60 (58.3)	51 (41.5)	6.32	0.016
	With family	43 (41.7)	72 (58.5)		
Education	No schooling	36 (35.0)	20 (16.3)	10.86	0.012
	Below middle school^b^	42 (40.7)	63 (51.2)		
	High school and above^c^	25 (24.3)	40 (32.5)		
Social activities	None	36 (35.0)	23 (18.7)	8.83	0.032
	Once a month	24 (23.3)	31 (25.2)		
	2-3/month	23 (22.3)	31 (25.2)		
	At least once a week	20 (19.4)	38 (30.9)		
Employment	No	90 (87.4)	93 (75.6)	5.04	0.028
	Yes	13 (12.6)	30 (24.4)		
Income level (10,000 KRW)	≤ = 50	54 (52.4)	38 (30.9)	14.31	0.004
	51–100	19 (18.4)	38 (30.9)		
	101–200	25 (24.3)	32 (26.0)		
	≥201	4 (4.9)	15 (12.2)		
Income source	Work	29 (28.1)	54 (43.9)	12.07	0.007
	Adult children	56 (54.4)	42 (34.2)		
	Property income	11 (10.7)	10 (8.1)		
	Other	7 (6.8)	17 (13.8)		

### Relationship of social network, health-promoting behaviors, and level of HRQOL to general characteristics

The descriptive statistics for the older adults with arthritis showed significant differences in social network, health-promoting behaviors, and all areas of HRQOL. Regarding age, the 74 or younger group showed higher levels of social network, health-promoting behaviors, and HRQOL. However, no age-related differences in HRQOL were found in older adults without arthritis. There was a gender-based difference in the level of HRQOL: males were found to have higher HRQOL than females. Both groups showed a significant difference in health-promoting behaviors and level of HRQOL depending on the presence of a cohabiting family. Older adults living with a family showed significantly higher health-promoting behaviors and HRQOL than older adults living alone. The arthritis group showed a larger difference than the group without arthritis in the level of HRQOL depending on the presence of a cohabiting family. Both groups showed a significant difference in social network, health-promoting behaviors, and HRQOL according to education, number of regular meetings, income level, and income source. Social network, health-promoting behaviors, and HRQOL were significantly lower for subjects who had no education, no regular meetings, and a monthly income of less than KRW 500,000. The arthritis group showed higher social network, health-promoting behaviors, and HRQOL when their source of income was work or real estate rather than their children (Table 2).

**Table 2:** Comparison of social network, HPLP, and level of HRQOL with general characteristics (N = 226).

Characteristics	Categories	Older adults with arthritis (n = 103)						Older adults without arthritis (n = 123)					
		Social network		HPLP†		HRQOL‡		Social network		HPLP†		HRQOL‡	
		M±SD	t or F (*p*) Scheffe's	M±SD	t or F (*p*) Scheffe's	M±SD	t or F (*p*) Scheffe's	M±SD	t or F (*p*) Scheffe's	M±SD	t or F (*p*) Scheffe's	M±SD	t or F (*p*) Scheffe's
Age (year)	≤ = 74	16.56±6.48	4.67 (< .000)	2.42±.57	4.06 (< .000)	.74±.15	3.82 (< .000)	18.37±5.79	3.82 (< .000)	2.65±.48	2.08 (.040)	.84±.17	1.36 (.176)
	≥75	10.36±6.86		1.89±.72		.63±.15		14.38±5.77		2.43±.66		.79±.21	
Gender	Male	12.33±7.19	-.57 (.573)	2.15±.72	.19 (.848)	.74±.17	1.80 (.082)	17.97±4.36	2.28 (.024)	2.62±.46	1.13 (.260)	.88±.16	3.23 (.003)
	Female	13.34±7.41		2.11±.70		.66±.16		15.40±6.73		2.49±.64		.77±.20	
Residing	Alone	9.71±6.34	-6.66 (.492)	1.76±.62	-7.61 (.040)	.59±.11	-7.01 (.001)	13.58±6.72	-4.51 (.008)	2.33±.65	-3.46 (.001)	.75±.23	-2.97 (.003)
	With family	17.90±5.89		2.62±.47		.79±.16		18.26±4.78		2.68±.48		.86±.15	
Education	No schooling^a^	6.50±3.94	42.20 (< .000) a<b<c	1.42±.30	56.66 (< .000) a<b,c	.56±.11	18.19 (< .000) a<b,c	9.15±5.85	22.63 (< .000) a<b<c	1.80±.49	26.70 (< .000) a<b,c	.61±.23	16.41 (< .000) a<b,c
	Below middle school^b^	15.83±5.85		2.48±.58		.72±.15		17.42±4.81		2.70±.43		.84±.16	
	High school and above^c^	18.16±6.58		2.53±.51		.77±.15		18.17±5.56		2.65±.56		.87±.15	
Social activities	None^a^	6.72±4.35	28.81 (< .000) a<b,c,d	1.51±.43	26.06 (< .000) a<b,c,d	.57±.10	12.67 (< .000) a<b,c,d	10.78±7.60	9.66 (< .000) a<b,c,d	1.96±.60	12.74 (< .000) a<b,c,d	.65±.26	10.25 (< .000) a<b,c,d
	1/month^b^	13.79±5.05		2.42±.68		.73±.16		17.39±4.59		2.76±.37		.89±.14	
	2-3/ month^c^	18.22±5.89		2.64±.44		.79±.17		18.26±5.50		2.73±.52		.88±.15	
	At least 1/week^d^	18.05±6.99		2.3±.60		.68±.16		17.24±4.77		2.57±.57		.80±.17	
Employ-ment	No	12.58±7.51	-2.06 (.193)	2.09±.74	-1.46 (.915)	.66±.16	-2.87 (.006)	15.97±6.42	-1.32 (.042)	2.54±.65	-.11 (.148)	.79±.21 .90±.14	-2.82 (.012)
	Yes	17.00±4.73		2.39±.27		.81±.17		17.43±4.90		2.55±.32			
Income (10,000 KRW)	≤ = 50^a^	8.87±6.12	20.63 (< .000) a<b,c,d	1.75±.69	17.71 (.041) a<b,c,d	.60±.13	14.19 (< .000) a<b,c,d	12.71±6.65	7.51 (< .000) a<b,c,d	2.32±.75	2.85 (< .000)	.73±.23 .77±.19 .91±.12 .94±.11	8.62 (< .000) a<c,d
	51–100^b^	16.74±5.08		2.29±.33		.71±.17		17.71±5.30		2.62±.53			
	101–200^c^	18.20±5.77		2.71±.47		.81±.14		18.09±4.32		2.67±.40			
	≥201^d^	20.20±5.89		2.63±.35		.80±.14		18.20±6.34		2.63±.49			
Income source	Work^a^	15.97±4.38	14.23 (< .000) a,c>b	2.47±.47	10.99 (< .000) a,c>b	.80±.16	23.29 (< .000) a,c>b	17.00±4.80	4.54 (.005) a<c	2.59±.43	5.58 (.001) b<c,d	.88±.16	5.89 (.001) a>b
	Adult children^b^	9.75±6.73		1.81±.67		.58±.11		13.88±7.07		2.28±.67		.72±.22	
	Property income^c^	21.36±6.89		2.64±.50		.74±.12		20.10±6.92		2.88±.57		.87±.15	
	Social security or other^d^	15.57±7.59		2.43±.89		.85±.16		18.00±4.81		2.80±.63		.82±.21	

†HPLP: Health Promotion Lifestyle

‡HRQOL: Health-Related Quality of Life

a, b, c, d: Post-hoc test using Scheffe's test

### Comparison of social network, health-promoting behaviors, and HRQOL between older adults with arthritis and without arthritis

The level of social network of the older adults with arthritis was 13.13±7.34 points out of a possible total of 30, which was lower than that of the older adults without arthritis (16.32±6.09 points). Both groups were found to have greater social engagement with family members than with friends. While the arthritis group scored lower for performing health-promoting behaviors, they obtained the highest scores in the stress management (2.27±.70) and nutrition (2.26±.66) domains. There was a significant difference of health-promoting behaviors between the older adults without arthritis and those with arthritis. Among the subcategory of health-promoting behaviors, ‘nutrition’ was only not significant factor between the two groups. The score of HRQOL ranges from -1, indicating serious problems to 1, indicating no problem; the group without arthritis obtained .82±.20, which was higher than the score obtained by the arthritis group, .68±.16 (Table 3).

**Table 3:** Comparison of social network, HPLP, and HRQOL between older adults with arthritis and without arthritis (N = 226).

Variables	Older adults with arthritis (n = 103)	Older adults without arthritis (n = 123)	Range	t	*p*
	Mean±SD	Mean±SD			
Social network	13.13±7.34	16.32±6.09	0~30	3.57	< .000
Family subscale	7.20±3.38	9.34±2.94	0~15	2.81	.007
Friend subscale	5.93±4.39	7.97±3.63	0~15	3.83	< .000
HPLP†	2.12±.70	2.54±.58	1~4	4.81	< .000
Self-actualization	2.12±.88	2.65±.73		4.86	< .000
Nutrition	2.26±.66	2.65±.64		4.43	< .000
Health responsibility	2.00±.76	2.32±.70		3.22	.001
Exercise	1.87±.82	2.32±.82		4.15	< .000
Interpersonal relationships	2.20±.76	2.63±.56		4.61	< .000
Stress management	2.27±.70	2.65±.61		4.26	< .000
HRQOL‡	.68±.16	.82±.20	-1~1	5.65	< .000

†HPLP: Health Promotion Lifestyle

‡HRQOL: Health-Related Quality of Life

### Correlations among social network, health-promoting behaviors, and level of HRQOL of older adults with and without arthritis

Correlation analyses of the relationship between social network, health-promoting behaviors, and HRQOL showed that social network and health-promoting behaviors were positively related to HRQOL for both groups. The older adults with arthritis showed the strongest correlations between social network and health-promoting behaviors (*r* = .67), followed by health-promoting behaviors and quality of life (*r* = .57), and social network and quality of life (*r* = .40). The older adults without arthritis showed the strongest correlations between social network and health-promoting behaviors (*r* = .53), followed by health-promoting behaviors and quality of life (*r* = .43), and social network and quality of life (*r* = .34) (Table 4).

**Table 4:** Correlations among social network, HPLP, and level of HRQOL of the older adults with and without arthritis (N = 226).

Variables	Older adults with arthritis (n = 103)		Older adults without arthritis (n = 123)	
	Social network *r*(*p*)	HPLP *r*(*p*)†	Social network *r*(*p*)	HPLP *r*(*p*)†
HRQOL‡	.40 (< .001)	.57 (< .001)	.34 (< .001)	.43 (< .001)
HPLP†	.67 (< .001)		.53 (< .001)	

†HPLP: Health Promotion Lifestyle

‡HRQOL: Health-Related Quality of Life

### Influencing factors of HRQOL

This study identified significant demographic variables in each group that are associated with health-related quality of life using *t*-tests and ANOVA. For the group of older adults with arthritis, all variables except gender were included as control variables, and for the group of older adults without arthritis. All variables except age were included. A multiple regression analysis was conducted with the general characteristic variables that were significantly different between the two groups. All variables were determined not to have the problem of multicollinearity since tolerance was 0.1 or greater and VIF was less than 10. The regression model for the older adults with arthritis was statistically significant (*F* = 40.71, *p* < .001), so factors that were associated with the HRQOL of older adults with arthritis were exercise and the presence of a cohabiting family, and the explanatory power of the two variables was 44%. The regression model for the older adults without arthritis was also statistically significant (*F* = 15.44, *p* = .001); among the subdomains of health-promoting behaviors (exercise), relationship with friends in the social network, and employment were associated with HRQOL, and the *R*^2^ was .26 (Table 5). When the stratified analysis was conducted using all older adults, both with and without arthritis, all variables were significantly associated with HRQOL. The results of the regression analysis indicated that exercise, employment, and education were significantly related to better HRQOL (Adj *R*^2^ = .38, *F* = 46.935, *p* <001). Using a regression, there was no interaction effect between social gathering and arthritis (t = .86, *p* = .39) on HRQOL. In the group of older adults with arthritis, the effect between gatherings and HRQOL was 0.053(t = 2.44, *p* < .001). For those without arthritis, the effect between gatherings and HRQOL was 0.035(t = 3.48, *p* = .015). Therefore the moderating effect of social gathering was significant in both groups, but the effect was small (Table 6).

**Table 5:** Associated factors of HRQOL.

	Variables	B	β	t	*p*	Adj *R*^2^	F
Older adults with arthritis	Exercise	.09	.43	4.69	< .001	.44	40.71 (< .001)
	Living with whom	.11	.32	3.46	.001		
				
Older adults without arthritis	Exercise	.08	.31	3.38	.001	.26	15.44 (.001)
	Social network (friend subscale)	.01	.22	2.37	.019		
	Employment	.09	.19	2.39	.018		

**Table 6:** Interaction effect between social gatherings and the older adults with and without arthritis.

Variables	β	SE	t	*p*	Adj *R*^2^	F
Arthritis	-.14	.06	-2.44	.02	.16	13.79 (< .001)
Social gatherings	.04	.01	2.44	.02		
Arthritis*Social gatherings	.02	.02	.86	.39		
Conditional effect of X on Y at value of the moderator (Arthritis)						
	Effect	se	t	*p*		
Without Arthritis	.03	.01	2.44	.02		
With Arthritis	.05	.02	3.48	< .001		

*: Interaction effect

## Discussion

The goal of the present study was to understand the relationship between the social networks and health-promoting behaviors of older adults with arthritis and without arthritis and to conduct a comparative analysis of the factors associated with HRQOL. There have been several Korean studies on the HRQOL of arthritic older adults, but the number of variables was limited. In addition, there has been a lack of studies considering the combination of social network and health-promoting behavior. Investigating the factors associated with HRQOL of older adults with arthritis, a chronic disease, is significant in understanding the health problems of a super-aged society. Also, there is the possibility of using the results as primary resources to build interventions to improve the well-being of older adults.

The results of the current study showed that the older adults with arthritis were more likely to include older adults living alone who were less educated and had no regular social gatherings compared to the older adults without arthritis. The HRQOL scores of the 74-or-younger age group were significantly higher than those adults 75-or-older for both older adults with arthritis and without; this finding was similar to other studies on chronic diseases [5,8,25]. Furthermore, the level of HRQOL was significantly lower for the group with arthritis than the group without arthritis; thus supporting the findings of previous studies [5,15].

For the older adults with arthritis, ‘no regular gathering’ was the most frequent response, while ‘regular gatherings once or more per week’ was the most frequent response for the older adults without arthritis. This finding is similar to that of Im’s study [26], who reported that having a limited social network is consistent with poor health status. For older adults, social gatherings are a way of maintaining social networks and exchanging information and are associated with the promotion of positive health behaviors [14]. The presence of arthritis was significantly related to HRQOL (F = 15.88, *p <* .*000*) as was social gathering was significantly related to HRQOL (F = 22.98, *p <* .*000*); however, the interaction of social gathering and arthritis was not significant (F = .86, *p* = .39). This result might be due to the relatively small sample size; thus, the finding that social gathering and arthritis were significantly associated with HRQOL but not the interaction. Further study, with a larger sample size, is needed to test this hypothesis. Observing regular activities or gatherings of older adults and providing opportunities for them to maintain social relationships with others could support improved HRQOL.

In the current study, the older adults with arthritis showed significantly different levels of HRQOL depending on their age; however, no difference was found for the older adults without arthritis. This is similar to the previous studies that found that older adults with arthritis reported lower quality of life than older adults without arthritis [27,28], the quality of life is worse as age increases [29]. We also need to consider HRQOL based on cultural background because family social support influences successful aging and quality of life in the Korean older adults [30,31]. Follow-up research is needed to compare the effect of age-stratified differences in HRQOL on adults with chronic diseases including arthritis and social support the consistency of the results.

No difference was found by gender in the older adults with arthritis, and the study by Kim [8] on osteoarthritis in older adults also found no difference by gender. The older adults without arthritis, however, showed gender differences in HRQOL; the HRQOL of male older adults was found to be higher than that of female older adults. This finding is supported by the studies of Kim [25] and Moon [17]. Older adults exchange social support and psychological stability with their family, but the results need to be interpreted taking into consideration the cultural differences in the male and female roles in Korea. The results in these studies may have been because Korea follows a patriarchal culture [26], and that female adults frequently play the role of caregivers for their spouses, children, or grandchildren rather than being care recipients [17]. In the 21^st^ century, however, social perceptions related to male and female roles are changing, and the provision of material or education related to role sharing for older adults will have a positive influence on the improvement of HRQOL.

The level of HRQOL was higher for older adults living with their family than for older adults living alone for both the older adults without arthritis and with arthritis. In particular, the difference in the score for quality of life in the presence of a cohabiting family was higher for the group with arthritis than the group without arthritis. In South Korea, family members who live with and take care of the older adult is considered as human resources to provide support for older adult’s daily lives and any difficulty with lives [32]. This finding is consistent with the findings of other studies on subjects with chronic diseases. For example, older adults who live alone reported poorer quality of life and life satisfaction compared to older adults living with others [17,25,32]. It can then be considered that the social and emotional support provided by the family is significantly associated with the quality of life of older adults. The social role formed through the relationship with family and society has an important meaning in the life of older adults. Research analyzing family support for older adults with arthritis through the analysis of HRQOL models suggests that the type of cohabitation of older adults is an important indicator that signifies the availability of human resources that can reduce the difficulties of daily living or role performance [32]. A review needs to be conducted on the possibility of providing nursing interventions and the scope of the interventions that can replace family support. In addition, the evaluation of a balanced exchange of support among cohabiting family members along with the investigation of the social role older adults play in the family rather than the mere presence of a cohabiting family appears to be needed to promote a better HRQOL for older adults.

The findings of the present study support the findings of previous studies [5,33] that the physical and emotional quality of life of older adults with arthritis is lower than that of older adults without arthritis because the social networks, health-promoting behaviors, and HRQOL of the older adults with arthritis was significantly lower than that of the older adults without arthritis. Both groups showed stronger family networks than networks with friends or neighbors; it can be considered that limitations in social activities due to retirement or health problems were a factor in shrinking the social network of older adults [10].

Social networks are closely related to health-promoting behaviors [34]. The relationship between social networks and health-promoting behaviors was stronger than the relationships between social networks and HRQOL or health-promoting behaviors and HRQOL in both groups, clearly indicating the importance of social networks in health-promoting behaviors. In particular, social isolation can be aggravated by physical disabilities or limitations in daily living caused by chronic diseases [6,7,35]. Since the importance of social support through family and friends on the quality of life of older adults with arthritis is greater than the individuals’ perception of health [36], the possibility of expanding social networks should be determined, and support systems should be formed to improve HRQOL.

Factors significantly associated with HRQOL of older adults with arthritis were exercise and the presence of a cohabiting family; the explanatory power of the two variables was 44%. The result suggests that continuing exercise and supportive relationships with cohabitants are vital for improving the HRQOL of older adults with arthritis [37] and that opportunities for older adults with arthritis to continue exercising need to be examined. Social support is an important influencing factor for the continuation of older adults’ exercise [38], and the provision of social support through the expansion of social networks is likely to have a positive effect on HRQOL along with health-promoting behaviors.

For older adults without arthritis, exercise, social networking with friends, and employment are significantly related to a better HRQOL with the explanatory power of 26.2%. The fact that the relationship between social networks with friends or neighbors and HRQOL is greater than that of the social network of family can indicate that the factors influencing HRQOL are changing with the times. This differs from the past when the family (e.g., spouse and children) was emphasized as the key factor for forming social networks, in the quality of life of older adults [25,39]. Asian culture is family-oriented; the family was considered to be the main social network for older adults in the past. For a long time, Confucianism has had a large influence on culture in Asian countries [40]. For example, Korean Confucianism emphasizes family relationships and serving one’s parents devotedly. The relationship of family impact on quality of life of older Korean adults has been clearly documented [41]. When the family has better relationships, the life satisfaction is higher among older Korean adults [31]. However, in modern times, social networks of friends or neighbors are one of the factors that are also associated with HRQOL, which is consistent with our findings. Similarly, older adults living with their spouse had higher HRQOL scores compared to older adults living alone. This might be the relationship between mutual social support from their family who live together and their HRQOL [17]. In addition, it can be considered that social responsibility is being emphasized for the improvement of HRQOL of older adults, and further research is necessary to explain differences in the results.

In this study, sociodemographic variables were not significantly related to HRQOL, unlike other research; for example, Moon’s study where income is considered as one of the socioeconomic factors that associated with health-related quality of life in older adults [17]. In the current study, however, exercise, one of the six subdomains of health-promoting behaviors for both groups, had the strongest association with better HRQOL. Thus, it is important to focus on physical health, and sustainable support for exercise programs, to maintain the HRQOL of older adults. In countries such as Japan and the U.S., research is ongoing on exercise programs for older adults, such as swimming programs for those with arthritis [37]. Efforts should be made to expand the application of exercise programs, that have examined through research, such as self-motivation programs [37,38] that increase adherence to exercise by older adults with arthritis. The implementation of exercise programs in which the health status and life pattern of older adults are considered, and exercise encouragement programs using artificial intelligence can act as important support systems that promote exercise among older adults. For example, when one considers today’s health conditions, there are applications to calculate the proper amount of exercise for the day; when one would like to work on certain parts of the body, the application shows appropriate exercises by body part and indicates exercise levels from mild to strenuous. Further, using an artificial intelligence program such as Siri, Google Assistant, or Alexa, regular alerts could be sent to encourage exercise. It is necessary to review the level of use of community facilities for older adults and consistently provide exercise programs and physical spaces that can serve as places where social activities can take place since the social engagement of older adults is affected by the physical environment, which further affects the structural characteristics of social networks [42].

Arthritis is a disease in which HRQOL can be improved by managing the symptoms of the disease through self-management along with medical treatments and maintaining and improving activities of daily life [8]. The present study revealed that, among various health promoting activities, exercise is significantly associated with HRQOL for older adults. Approaches to the improvement of HRQOL of each individual should be tailored to the individual depending on their health status. Close attention should be paid to applying the findings of the present study to all older adults since the subjects were older adults residing in a local area. Likewise, because HRQOL was analyzed by classifying subjects based on self-reports of an arthritis diagnosis, care should be used when interpreting this study’s findings. In this research, we focused on the relationship of social networks and health-promoting behaviors on HRQOL of older Korean adults with arthritis and without arthritis, possibly limiting the generalizability of the findings to older adults from another ethnic group.

Furthermore, we used a cross-sectional design, so it was not possible to test causal-effect relationships. Thus, a longitudinal-design would be helpful to understand across relationships among social networks, health-promoting behaviors, and quality of life. Using stratified analysis requires having a larger sample size to decrease the probability of Type II error and increased reliability. This research, however, is a secondary data analysis using a subset of data from a previous study. While the sample size used was deemed sufficient by calculating the G-power, we should be cautious when generalizing this finding.

## Conclusions and recommendations

Social networks, health-promoting behaviors, and quality of life were found to be lower for older adults with arthritis than older adults without arthritis. Significant differences were found in the level of HRQOL between older adults with and without arthritis according to age, presence of a cohabiting family, educational level, number of regular gatherings, employment, income level, and income source; for both groups, exercise, one of the subdomains of health-promoting behaviors, was significantly related to their HRQOL.

Based on the results of the present study, the following suggestions are made.

First, institutional efforts are necessary to expand not only the scope of the personal networks of older adults with arthritis but also their social relationships because social networks were found to be one of the important factors associated with quality of life. Second, there should be an evaluation of the quality of social support, rather than relying on the presence of a cohabiting family, to promote the HRQOL of older adults. Third, while financial support or the engagement of social networks are important for the improvement of the HRQOL of older adults, support for consistently sustainable exercise programs appears to be even more desirable.
